# Editorial: De-escalating Threat: The Psychophysiology of Police Decision Making

**DOI:** 10.3389/fpsyg.2020.01112

**Published:** 2020-05-26

**Authors:** Judith P. Andersen, Eamonn P. Arble, Peter Ian Collins

**Affiliations:** ^1^Department of Psychology, University of Toronto Mississauga, Mississauga, ON, Canada; ^2^Department of Psychology, Eastern Michigan University, Ypsilanti, MI, United States; ^3^Division of Forensic Psychiatry, University of Toronto, Toronto, ON, Canada

**Keywords:** police, de-escalation, psychophysiology, decision making, stress

Police officers are required to make rapid, high-stakes decisions on a routine basis. Scientific research on making decisions during situations that are perceived as threatening is situated in the context of neurological and physiological processing (LeDoux and Pine, 2016). However, despite advances in the empirical understanding of decision making during high-stakes events, popular (“layperson”) discussion surrounding police decision making has not been readily informed by contemporary research on neurological processing of threat or psychophysiological reactivity to stress. Rather, social debate tends to focus on psychosocial issues, including the narrative that police attitudes and beliefs are the central factors directing police behavior (e.g., Phillips and Sobol, 2012).

The goal of this Frontiers Research Topic: “*De-escalating Threat: The Psychophysiology of Police Decision Making*,” is to bring awareness to the emerging theoretical and empirical examination of police decision making, including neuroscience and psychophysiological perspectives. Theoretical models and recent research indicate that performance during cognitively demanding tasks is related to a psychophysiological feedback network comprised of bidirectional signals between the following:

- Higher order cognitive and emotional processing regions of the brain (e.g., prefrontal cortex (PFC) and limbic system),
- Subcortical regions of the central autonomic network (e.g., brainstem),
- The peripheral autonomic nervous system (ANS) (e.g., parasympathetic and sympathetic), including the heart and lungs (Thayer et al., 2009; LeDoux and Pine, 2016; Smith et al., 2017; Arpaia and Andersen, 2019).

Thus, from theoretical and empirical perspectives, the research within this special edition will consider how the functioning of the bidirectional signals between the brain and the central and peripheral nervous systems relate to police decision making in order to inform police training, policy, and legal judgements regarding police behavior.

All of the articles in this special section can be placed into one of three groupings. In the first set of articles, the authors demonstrate that psychophysiological stress can be measured in field settings and that these biometric indices are associated with important indicators of skill performance, situational factors, and individual characteristics that interact to influence police decision making. Two critical findings emerge from this research. First, the variables identified in these investigations appear malleable by a number of factors, most importantly, training history and psychophysiological readiness to perform occupational tasks (Arble et al.). Second, traditionally it was thought that higher arousal always equated with poorer performance due to the potential for cognitive, perceptual, and sensory distortions observed among humans under extreme threat. For example see Giessing et al. in which the authors examine the level of impairment in shooting skills associated with high stress, anxiety-producing scenarios. However, other articles in this issue identify evidence that physiological arousal during threat may impact police skills differentially. Specifically, Arble et al. examined police simulations of life-threatening events and found that physiological arousal was associated with detriments in verbal communication but not tactical or non-verbal “automatic” skills. Baldwin et al. found arousal was influenced differentially by call type (e.g., priority, weapon present) and stage (e.g., briefing, *en route*) among active duty officers on real calls. Furthermore, Anderson et al. and Bertilsson et al., highlight that stress related biochemicals and ANS arousal impacts police differentially, sometimes to the officers' benefit and others to the detriment of skills. In light of these findings, implications for police training and evaluation are explored.

In a second set of articles, authors apply theory drawn from studying civilian behavior to that of police decision making. The biopsychosocial model (BPS) is used to develop an understanding of how police officer evaluations of occupational challenges, and their ability to overcome them, impact their performance (Kelley et al.). Similarly, the Theory of Constructed Emotion (TCE) (Barrett, 2016) is used as a platform to characterize the neurobiological mechanisms underlying adaptive police decision-making (Fridman et al.). Specifically, Fridman et al., articulate a critical role played by the ANS in regulating the short-term energy expenditures that are crucial to performance success in the high-pressure context police officers are expected to operate within. Across these articles, theoretical application is accompanied by the understanding that any actual police encounter has a wide range of variables that influence the outcome of the event (e.g., whether a shooting happens or not) and that these variables are very different in the real world as compared to laboratory settings. Thus, the authors propose important areas for future investigation to demonstrate the viability of these theories within the context of police field work.

In a third set of articles, authors review findings from experimental studies to better understand the effect of complex motor learning and psychophysiology on risk assessment and decision making. Harman et al. apply basic findings from experimental studies in judgment and decision making to posit that reactions to risk and threat are strongly influenced by previous decisions made in similar contexts (e.g., police experience on the road, in training, or even through media exposure). Di Nota and Huhta provide a synthesis of evidence from fields including cognitive psychology, adult education, clinical neuroscience, and applied psychophysiological research as it pertains to police training. The review concludes with concrete recommendations to promote evidence-based practices for training skills essential to modern policing, including physical use of force tactics, verbal communication, situational awareness, and high-stakes decision making. The insights across these articles provide a direct translation of current scientific knowledge into actionable recommendations for existing training policies and procedures that serve to protect the safety of both police and the general public.

The articles in this special section utilize evidence-based approaches to probe empirical and theoretical questions related to de-escalating high-stakes situations, and informed decision making regarding the use of force, skills training and motor learning. The articles in the section highlight the need to empirically test theoretical models under ecologically valid conditions (Andersen et al., 2018).

## Author Contributions

JA and EA drafted the manuscript. PC provided conceptual and editorial contributions. All authors contributed to manuscript revision, read, and approved the submitted version.

## Conflict of Interest

The authors declare that the research was conducted in the absence of any commercial or financial relationships that could be construed as a potential conflict of interest.
